# Association synchrone d'un cancer du sein bilatéral et d'une tumeur stromale gastro-intestinale: à propos d'un rare cas

**DOI:** 10.11604/pamj.2015.20.45.5771

**Published:** 2015-01-16

**Authors:** Mohammed Afif, Imane Ouziane, Fadila Kouhen, Jihane Khalil, Fedwa Elomrani, Hanan Elkacemi, Tayeb Kebdani, Hassan Errihani, Noureddine Benjaafar

**Affiliations:** 1 Service de Radiothérapie, Institut National d'Oncologie, Université Mohammed V, Rabat, Maroc; 2 Service d'Oncologie Médicale, Institut National d'Oncologie, Université Mohammed V, Rabat, Maroc

**Keywords:** Cancer du sein, tumeur stromale, association, rare, breast cancer, stromal tumor, association, rare

## Abstract

Les tumeurs stromales gastro-intestinales sont les tumeurs mésenchymateuses les plus fréquentes, pourtant, leur association avec les tumeurs du sein sont rares, seulement quelques cas cliniques sont rapportés dans la littérature. Nous rapportons l'observation d'une jeune femme de 41 ans, suivie à l'institut national d'oncologie de Rabat, pour un carcinome canalaire du sein, bilatéral, chez qui le bilan d'extension a objectivé une tumeur stromale de type gastro-intestinale aux dépens de l'estomac. Nous décrivons à travers cette observation les aspects épidémiologiques, cliniques, et les particularités de la prise en charge de cette association rare.

## Introduction

Les associations synchrones de deux ou plusieurs tumeurs sont rares, et posent de nombreuses difficultés diagnostiques et thérapeutiques. Les associations du cancer du sein avec les tumeurs stromales gastro-intestinales (GIST) sont exceptionnelles, l'analyse des données de la littérature rapporte quelques cas isolés, à notre connaissance, seulement deux cas d'association synchrone d'un cancer du sein bilatéral et d'une GIST ont été rapportés jusqu’à nos jours [1, 2].

## Patient et observation

**Figure 1:**
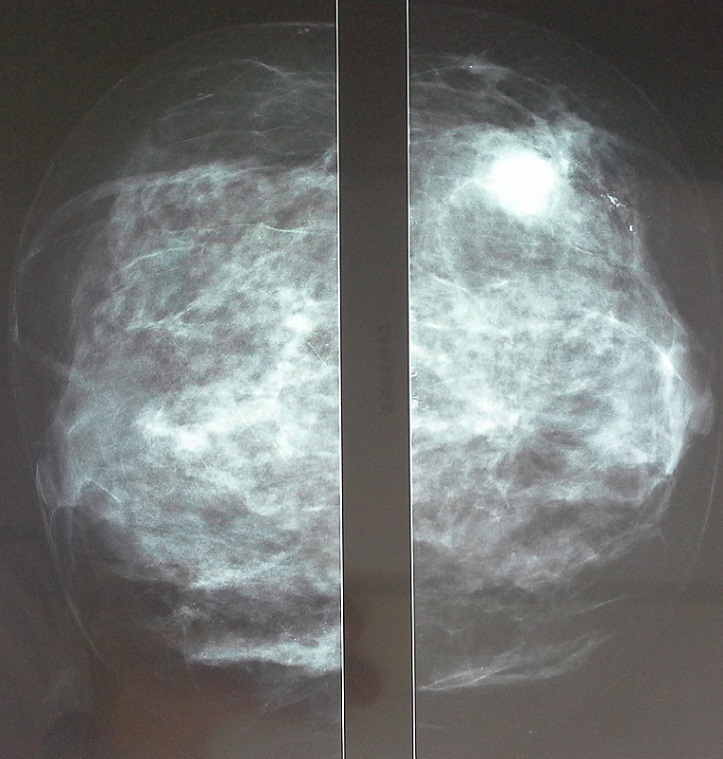
Mammographie montrant un nodule au niveau du quadrant supero-externe du sein droit, ACR V

Il s'agit d'une patiente âgée de 41 ans, sans antécédents pathologiques particuliers, non ménopausée, qui a présenté un nodule au niveau du sein droit. La mammographie associée à l’échographie mammaire a objectivé deux nodules suspects, l'un siégeant au niveau du quadrant supéro-externe du sein droit (Figure 1), et l'autre au niveau du sein gauche, de siège rétro-mamelonnaire. La patiente a bénéficié d'une mastectomie droite et zonectomie gauche, avec un curage ganglionnaire bilatéral, l’étude histologique a montré: à droite: un carcinome canalaire infiltrant, de grade SBR II, sans emboles vasculaires, Ki 67 à 15%, limites saines, curage ganglionnaire négatif (14 ganglions négatifs), récepteurs hormonaux positifs (RE = 0%, RP= 70%), et statut HER2 négatif, cette lésion a été classée pT2N0; à gauche: un carcinome canalaire, SBR II, sans emboles vasculaire ni lymphatiques, curage ganglionnaire négatif (9 ganglions négatifs), cette tumeur était classé pT1N0, les récepteurs hormonaux étaient fortement exprimés (RE= 70%, RP= 100%), et le statut HER2 négatif.

**Figure 2:**
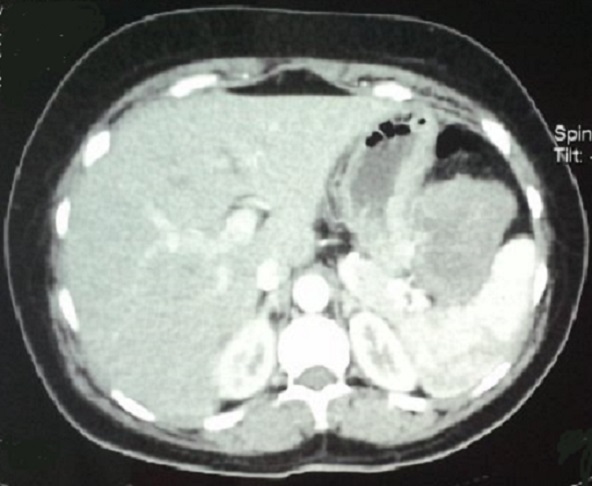
Image de la TDM Abdominale en coupe axiale, demontrant la masse gastrique suspecte de GIST gastrique

Le bilan d'extension réalisé en post opératoire incluant une échographie abdominale avait objectivé une masse épigastrique, la tomodensitométrie (TDM) abdominale a confirmé la présence d'un processus tumoral tissulaire, siégeant au niveau de l'espace inter-gastro-splénique, en contact intime avec l'estomac, bien limité, de contours lobulés, mesurant 94x86mm (Figure 2). Le reste du bilan d'extension notamment la scintigraphie osseuse était sans particularités. Une gastrectomie atypique emportant la tumeur a été réalisée 1 mois après la chirurgie mammaire, et l’étude histologique de la tumeur avait objectivé une prolifération tumorale fusiforme, en faveur d'une tumeur stromale de type gastro-intestinale (GIST), de haut risque selon Fletcher, exprimant les CD34, et CD 117 à l’étude immunohistochimique.

**Figure 3:**
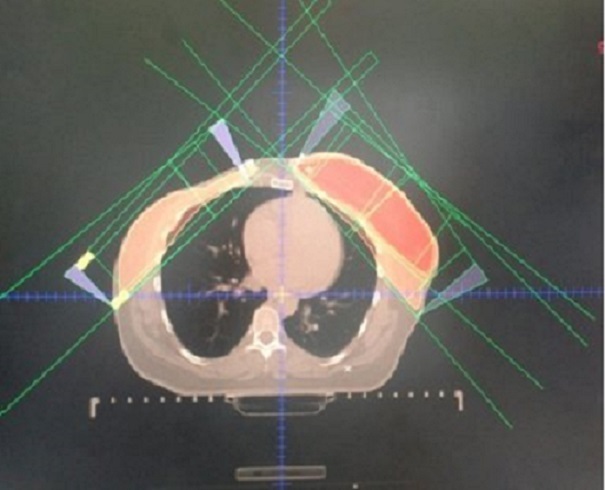
Image système de planification du traitement, objectivant la balistique utilisée au cours de l irradiation mammaire chez notre patiente

La patiente a reçu en postopératoire une chimiothérapie de type séquentielle à base d'anthracyclines et docetaxel, suivie une radiothérapie adjuvante sur la paroi thoracique droite, et le sein gauche, à la dose de 50 Gy, en fractionnement classique, en utilisant des photons X de 6MV, par des champs tangentiels (Figure 3), suivie d'une surimpression sur le lit tumoral gauche à la dose de 12 Gy, l’étalement était de 48 jours. Actuellement la patiente est sous imatinib, à la dose de 400mg/ jour, et une hormonothérapie à base du Tamoxifène, à la dose 20mg/ jour, avec bonne tolérance clinique et biologique, 18 mois après la chirurgie de la GIST.

## Discussion

L'association des tumeurs stromales gastro-intestinales (GIST) avec d'autres tumeurs primitives est rare, elle se voit essentiellement avec des tumeurs du tractus digestif (47% des cas d'associations) [2]. L'association avec le cancer du sein est très rare et se voit dans moins de 7% des cas [3]. Les mécanismes expliquant cette association ne sont pas bien élucidés, l'implication de facteurs génétiques a été évoquée par certains auteurs [3, 4], mais aucun n'a pu la confirmer vu son caractère exceptionnel. La majorité des cas publiées ont a été rapportés chez des patientes porteuses de neurofibromatose de type 1 (neurofibromatose de Von Recklinghausen). L'incidence des GIST chez les patients atteints de NF-1 varie entre 3,9% et 25% [5, 6]. Les GIST associées à la neurofibromatose sont en général de bon pronostic, et la mutation c-KIT est plus rare, ainsi, le mécanisme de développement de ces tumeurs stromales peut s'expliquer par la perte d'hétérozygotie de NF-1, plutôt que d'une mutation du gène c-KIT [2], notre patiente ne présentait pas de neurofibromatose.

Cette association est plus souvent métachrone, et en principe, le cancer du sein précède le diagnostic de GIST. L'association GIST avec d'autres tumeurs est de plus en plus fréquente que les patients vivent plus longtemps après le diagnostic d'autres cancers [7]. La particularité de notre observation réside dans le fait que le diagnostic de GIST à été fait de façon synchrone avec celui du cancer du sein bilatéral, et la patiente a pu bénéficier des traitements des deux tumeurs de façon simultanée. La prise en charge des patientes présentant une tumeur du sein associée à une GIST rejoint celle des malades ayant une double localisation tumorale. Dans notre cas, la patiente a bénéficié de chirurgie mammaire suivie d'exérèse de la tumeur gastrique; les traitements adjuvants ont été démarrés après chirurgie de la tumeur stromale, l'Imatinib a été démarré après la chimiothérapie et la radiothérapie adjuvante afin d’éviter les interactions médicamenteuses. Cette prise en charge n'est pas consensuelle vu la rareté des cas décrits. Le traitement médical des GIST peut interférer avec l'hormonothérapie du cancer du sein. En effet, l'imatinib peut réduire l'efficacité du Tamoxifène par diminution de la production de son métabolite actif par inhibition des enzymes CYP 2D6 and 3A4 [8], pourtant, cet effet est réduit, et il n'est pas recommandé d'augmenter les doses de tamoxifène.

## Conclusion

Il est difficile d'objectiver une relation, notamment génétique, entre le cancer du sein et les GIST sur de simples cas cliniques. Des études supplémentaires sur un nombre plus important de malades sont nécessaires afin de confirmer le lien entre les deux tumeurs, ainsi que tout rôle éventuel des mutations de c-kit dans la genèse des cancers du sein.
